# Oxidative Stress-Induced DNA Damage and Repair in Human Peripheral Blood Mononuclear Cells: Protective Role of Hemoglobin

**DOI:** 10.1371/journal.pone.0068341

**Published:** 2013-07-09

**Authors:** Anat Gafter-Gvili, Boris Zingerman, Benaya Rozen-Zvi, Yaacov Ori, Hefziba Green, Ido Lubin, Tsipora Malachi, Uzi Gafter, Michal Herman-Edelstein

**Affiliations:** 1 Department of Nephrology & Hypertension, Rabin Medical Center, Petah Tikva, Israel; 2 Department of Medicine E, Rabin Medical Center, Petah Tikva, Israel; 3 Felsenstein Medical Research Institute, Rabin Medical Center, Petah-Tikva, Israel; 4 Sackler School of Medicine, Tel-Aviv University, Tel-Aviv, Israel; Institute of Molecular Genetics IMG-CNR, Italy

## Abstract

**Background:**

DNA repair is a cellular defence mechanism responding to DNA damage caused in large part by oxidative stress. There is a controversy with regard to the effect of red blood cells on DNA damage and cellular response.

**Aim:**

To investigate the effect of red blood cells on H_2_O_2_-induced DNA damage and repair in human peripheral blood mononuclear cells.

**Methods:**

DNA breaks were induced in peripheral blood mononuclear cells by H_2_O_2_ in the absence or presence of red blood cells, red blood cells hemolysate or hemoglobin. DNA repair was measured by ^3^H-thymidine uptake, % double-stranded DNA was measured by fluorometric assay of DNA unwinding. DNA damage was measured by the comet assay and by the detection of histone H2AX phosphorylation.

**Results:**

Red blood cells and red blood cells hemolysate reduced DNA repair in a dose-dependent manner. Red blood cells hemolysate reduced % double-stranded DNA, DNA damage and phosphorylation of histone H2AX. Hemoglobin had the same effect as red blood cells hemolysate on % double-stranded DNA.

**Conclusion:**

Red blood cells, via red blood cells hemolysate and hemoglobin, reduced the effect of oxidative stress on peripheral blood mononuclear cell DNA damage and phosphorylation of histone H2AX. Consequently, recruitment of DNA repair proteins diminished with reduction of DNA repair. This suggests that anemia predisposes to increased oxidative stress induced DNA damage, while a higher hemoglobin level provides protection against oxidative-stress-induced DNA damage.

## Introduction

The DNA repair system is a cellular defence mechanism responding to DNA damage caused in large part by oxidative stress. The DNA damaging agents are either external, such as pollution and exposure to sunshine or other types of irradiation, or internal such as a consequence of replication or metabolic pathways that produce reactive oxygen species (ROS). One of the ROS which causes DNA damage is H_2_O_2_. H_2_O_2_ induces double strand breaks (DSBs), which are followed by DNA repair [1], [2]. The H_2_O_2_-induced DNA repair measured in peripheral blood mononuclear cells (PBMC) has been previously used in our laboratory [1], [3], [4]. Phosphorylation of the histone H2AX is an early response to DNA damage, especially DSBs. It is the first step in recruiting DNA repair proteins to the DSBs site [5].

It has been observed that red blood cells (RBC) may affect the DNA damage and repair system. The active substance may be either the whole hemoglobin molecule or the ferrum component or other metabolites, such as adenosine phosphate, which exist in RBC. The published effects of whole RBC or RBC hemolysate are contradictive: some investigators did not find any association between DNA damage and hemoglobin [6], others found that hemoglobin caused DNA damage similar to the effect of H_2_O_2_ [7], [8], and another group found a negative correlation between hemoglobin level and DNA damage [9]. Porto et al showed that RBC protected cultured lymphocytes against chromosome breakage induced by the alkylating agent diepoxybutane. This effect was attributed to the RBC hemolysate and hemoglobin, while RBC membrane had no effect [10].

The objectives of our study were: (1) to measure the effect of autologous RBC and RBC hemolysate on DNA repair ability of PBMC stimulated in vitro by H_2_O_2_; (2) to determine the effect of RBC hemolysate and hemoglobin on % double-stranded DNA as a measure for DNA damage by H_2_O_2_. A higher level of % double-stranded DNA indicates less DNA breakage; (3) to assess the effect of RBC hemolysate on H_2_O_2_-induced phosphorylation of histone H2AX in PBMC; (4) to evaluate the effect of RBC hemolysate on H_2_O_2_-induced DNA breaks in PBMC by the comet assay.

## Methods

The study was approved by the Rabin Medical Center Institutional Review Board. The whole study was conducted in our center in Petah Tikva, Israel. We did not study humans, but used blood cells, which were purchased from the local blood bank. Hence the Review Board waived the need for informed consent.

### Blood

Buffycoats of fresh blood from apparently healthy donors were obtained from the local blood bank. The buffycoat, which was enriched with white blood cells (WBC) but contained also RBC, was divided into two portions.

### PBMC

Cells were separated from one portion by histopaque gradient centrifugation. In order to eliminate RBC contamination, the PBMC underwent a procedure of RBC hemolysation as follows: cell pellet was thoroughly mixed with 10.4 ml 0.24% NaCl hypotonic solution for 20 seconds, followed by 0.88 ml 8.7% NaCl hypertonic solution and diluted to 50 ml with PBS. After centrifugation this procedure was repeated once more. PBMC were suspended in RPMI-1640 complete medium and counted in a Neubauer chamber. DNA repair ability was measured at once. Cells for ds-DNA measurements, phosphorylation of histone h2AX and the comet assay were kept overnight at 4°C in complete RPMI medium and were processed the next morning.

### RBC Hemolysate

The other portion of the buffycoat unit was diluted with PBS and centrifuged at room temperature for 10 minutes at 3000 rpm. Supernatant and the upper layer of white blood cells were removed by suction and the washing procedure of the RBC pellet was repeated twice more until 5 ml of packed red cells were obtained. Five ml of 0.24% NaCl hypotonic solution were added to the packed cells, thoroughly mixed for 1 min and centrifuged for 20 minutes at 3000 rpm. Three ml of clear hemolysate were carefully collected from the upper layer and a sample of it was hematologically analysed. The RBC hemolysate added to the various experiments contained only traces of WBC. Osmolarity of the hemolysate was also measured and crystalline NaCl was dissolved in the hemolysate to reach iso-osmolarity. RBC hemolysate was added to the DNA repair, which was measured the same day, or kept overnight at 4°C and added to ds-DNA measurement, H2AX phosphorylation and the comet assay the next day according to protocols. The RBC hemolysate was added to the reaction mixture at various volumes. The highest concentration of hemolysate was 2% ^v^/_v_ per reaction. As the hemolysate was always prepared exactly by the same methods, the range of the hemoglobin level in the hemolysate was small. Thus, 2% ^v^/_v_ RBC hemolysate contained 2.00±0.07 mg hemoglobin per ml reaction medium.

### DNA Repair

We assessed the effect of both RBC and RBC hemolysate on DNA repair. DNA repair ability was measured in H_2_O_2_-stimulated PBMC by the method of ^3^H-thymidine incorporation, as previously described [1], [11] with modifications. The assay was performed in quadruplicates of 2×10^6^ PBMC per tube in 1.25 ml complete RPMI-1640 medium supplemented with 1% glutamine, 1% antibiotic solution (streptomycin, penicillin and amphotericin B), 1 mmol/L CaCl_2_, 0.1% bovine serum albumin and 10 mmol/L hydroxyurea (which inhibits scheduled DNA replication). Blank tubes contained no cells. Tubes were kept in an ice bath and then either packed RBCs (3% or 8% ^v^/_v_) or RBC hemolysate were added to produce a dose-response concentration of 0%, 0.3%, 0.6%, 1% and 2% ^v^/_v_ hemolysate in the reaction mixture. Uniformity of the volume in the reaction tubes was achieved by the addition of saline. Tubes with neither packed RBCs nor RBC hemolysate (0%) were referred to as control. Ten µl of H_2_O_2_ (final concentration of 200 µmol/L) were added to enable determination of H_2_O_2_-induced DNA repair. The tubes were transferred to a shaking water bath at 37°C for 60 seconds and then to an incubator at 37°C with a humid atmosphere of 5% CO_2_ for 30 minutes. The tubes were cooled on ice, 10 µl of 1 µCi/ml 3H-thymidine final concentration was added to all the tubes and incubation at 37°C continued for 120 minutes. The tubes were cooled, diluted with cold saline, washed several times with cold saline to remove surplus of radioactivity from the supernatant, and then nuclei-incorporated radioactivity was measured after lysis with NaOH, sedimentation with TCA, filtration through glass fibre filters and washing with plenty of 96% Ethanol. cpm were counted in scintillation fluid. H_2_O_2_-induced DNA repairwas expressed as cpm of nuclei-incorporated ^3^H-thymidine per 2×10^6^ cells, after subtracting cpm of parallel blanks without H_2_O_2_.

### Double-stranded DNA

ds-DNA was determined by fluorometric assay of DNA unwinding (FADU) method (12). PBMC which were kept overnight in complete medium were centrifuged at 4°C, suspended in cold myoinositol buffer pH 7.2, and divided between reaction tubes, so that all the tubes contained an equal number of cells (1–5×10^6^ cells/1.06 ml per tube). PBMC were incubated in the presence of increasing concentration of H_2_O_2_ as follows, 10, 20, 50, 100 and 200 µmol/L, and ds-DNA was determined. Based on the dose-effect in all the following experiments, we used 200 µmol/L. Ten µl of H_2_O_2_ (200 µmol/L end concentration) were added to the reaction mixture, except for tubes in which basal %ds-DNA was measured. ds-DNA was determined in PBMC in the presence of RBC hemolysate, at the following concentrations: 0%, 0.3%, 0.6%, 1% and 2% ^v^/_v_ RBC hemolysate in the reaction, which lasted 40 minutes in a shaking water bath at 37°C, prior to ds-DNA measurement. In some experiments human hemoglobin was added instead of hemolysate at a concentration of 1.8 mg/ml reaction buffer, which is similar to its concentration in 2% ^v^/_v_ hemolysate. After the reaction, FADU was performed as follows: for each hemolysate concentration three conditions were performed in quadruplicates of 0.2 ml of the reaction mixture: total ds-DNA (T), blanks (B) and partial ds-DNA (P), in which T retained natural DNA, in B double strands of DNA were decomposed by sonication and in P a partial unwinding was achieved by the alkaline unwinding condition [12]. The method is based on the assumption that the more DNA breaks are present in the molecule, the faster is its unwinding in alkaline condition. The unwinding reaction was performed in strictly regulated pH, time and temperature. After preparing the three sorts of cells for each hemolysate concentration, ds-DNA was determined by the fluorescent dye ethidium bromide at a wave length of 520 nm excitation and 590 nm emission. % ds-DNA was calculated according to the equation % ds-DNA = (P-B)100/T-B, in which P,B and T were cps of fluorescence of partial unwindings, blanks and totals ds-DNA, respectively (12). Recently, an automated version of FADU analysis was published [13]–[15] and we used the equation of this calculation to compare between samples as follows: ds-DNA ratio = (P_2-_B_2_/T_2_-B_2_)/(P_1-_B_1_/T_1_-B_1_), where P_2_, B_2_ and T_2_ are fluorescence counts of P,B and T of the experimental tubes with RBC hemolysate, while P_1_, B_1_ and T_1_ are the fluorescence counts of the control tubes without RBC hemolysate. The value of P_1_-B_1_/T_1_-B_1_ was constant in every experiment. In all the experiments, the calculated control ds-DNA = 1.00 and the experimental results are expressed as ratios of the control, whereas in the original paper [12] the results were expressed as %ds-DNA, which varied also between controls.

### Detection of H2AX Phosphorylation

Following incubation of PBMC with H_2_O_2_, the cells were washed with PBS, then fixed and permeabilised using H2AX phosphorylation assay kit (Millipore, USA) according to the manufacturer’s protocol [16], [17].

### Comet Assay

DNA damage was determined by the comet assay (single-cell gel electrophoresis) using the comet assay kit (Cell Biolab, USA) according to the manufacturer’s protocol. Electrophoresis was run in an alkaline buffer [8].

### Solutions

For DNA repair analysis 0.5 N NaOH was used for hemolysis after ^3^H-thymidine incorporation, 0.5 N HCl was used for neutralisation after hemolysis and 10% TCA solution was used for DNA sedimentation. For ds-DNA analysis human hemoglobin was freshly dissolved before incubation. Myoinositol buffer, 9 M urea, DNA disruption solution, equilibrated NaOH solutions for exact pH control and ethidium bromide solution for fluorescence spectrometry were prepared as instructed in Methods in Enzymology [12].

### Materials

^3^H–thymidine, specific activity 40–60 Ci/mmol, was purchased from Perkin Elmer, Boston, USA. Medium ingredients were purchased from Biological Industries, Beit Ha’emek, Israel. Human hemoglobin was purchased from Sigma, Rehovot, Israel. Scintillation liquid Ultima Gold was purchased from Perkin Elmer, Waltham MA, USA. Other chemicals were purchased from Sigma, Rehovot, Israel.

### Instruments

Radioactivity was counted in a LKB 1217 Rackbeta liquid scintillation counter, Wallac, Finland. Fluorescence was measured in disposable cuvettes in a Ratio Master Fluorescence spectrometer, Photon Technology International, USA.

### Statistical Analysis

Data are presented as mean ± SE. Student’s t test for paired data was used for comparisons, unless otherwise specified. Two tailed p<0.05 was considered significant.

## Results

### Effect of H_2_O_2_ on ds-DNA

**Figure 1:**
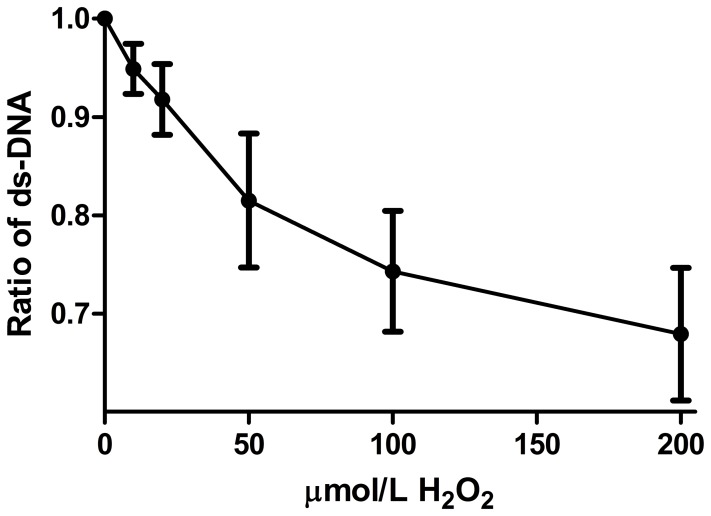
Dose response curve of the effect of H_2_O_2_ on ds DNA. Effect of 10, 20, 50, 100 and 200 µmol/L H_2_O_2_ on PBMC was tested: 100 and 200 µmol/L reduced %ds DNA significantly (p<0.02, p<0.01, respectively).

A dose effect of H_2_O_2_ on ds-DNA in PBMC was measured. As illustrated in Fig. 1 (N = 4), there was a progressive reduction in %ds-DNA, as the concentration of H_2_O_2_ rose from10 µmol/L to 200 µmol/L. Compared to control, 100 and 200 µmol/L of H_2_O_2_ reduced %ds-DNA significantly (p<0.02, p<0.01, respectively). We did not use a concentration higher than 200 µmol/L, since we had previously observed that a higher concentration affected cell viability.

### Effect of RBC on DNA Repair

H_2_O_2_-induced DNA repair without addition of RBC was 998.9±18.1 (N = 4) and was defined 100%. The addition of 40 µl packed RBC reduced DNA repair by 57.3% to 426.8±39.5 cpm/2×10^6^ (N = 4, p<0.001).With the addition of 100 µl, RBC DNA repair further decreased by 74.2% to 258.1±24.3 cpm/2×10^6^ cells (N = 4, p<0.001).

### Effect of RBC hemolysate on DNA Repair

**Figure 2:**
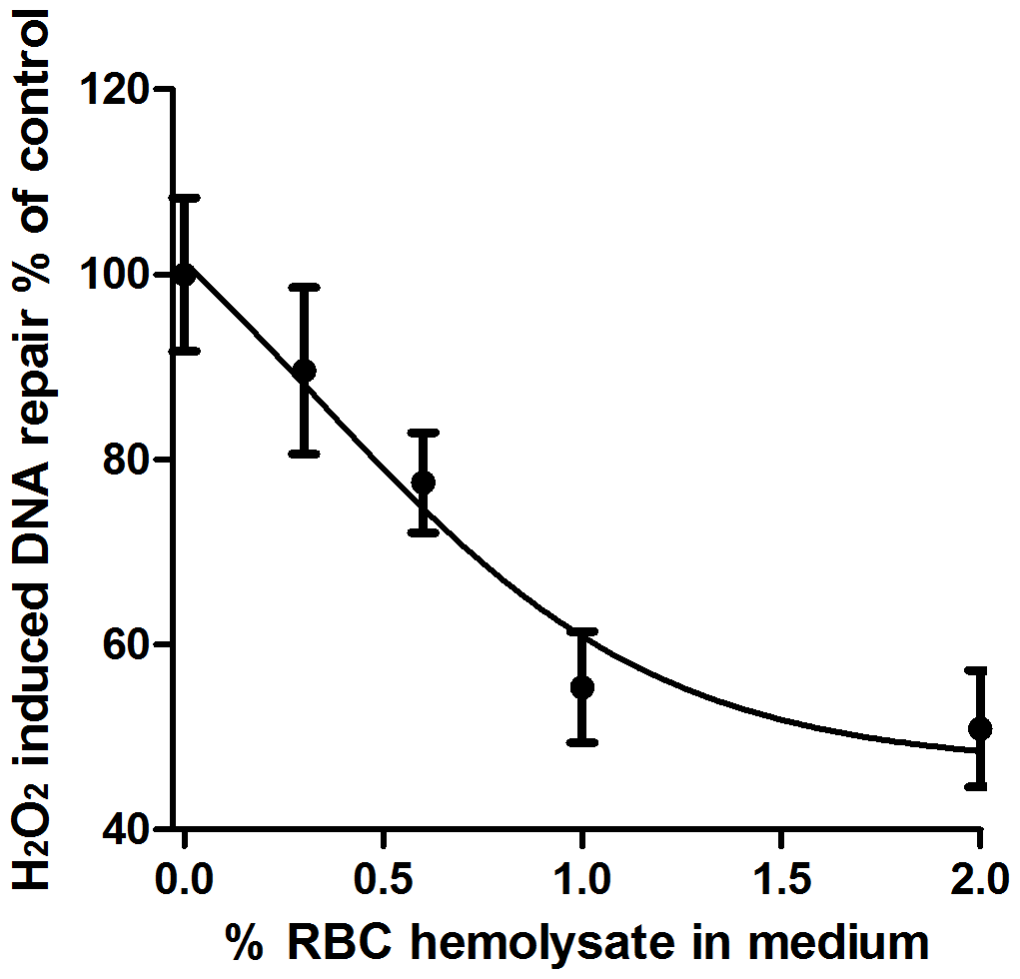
Dose-response curve of the effect of RBC hemolysate (% ^v^/_v_ in medium) on H_2_O_2_-induced DNA repair (% of control) in PBMC. For the effects of 0.3, 0.6, 1 and 2% ^v^/_v_ hemolysate, p = NS, p = NS, p<0.005 and p<0.005, respectively.

RBC hemolysate reduced H_2_O_2_-induced DNA repair in a dose-dependent manner. Tubes with H_2_O_2_ but without hemolysate served as a control group and its DNA repair ability was defined 100% (N = 8). Without hemolysate DNA repair ability was 100±8.03%.Addition of various volumes of hemolysate into the reaction medium reduced DNA repair ability as follows: 0.3%, 0.6%, 1% and 2% ^v^/_v_ produced DNA repair ability of 84.6±9.00% (p = NS ), 77.5±5.43% (p = NS), 55.4±5.95% (p<0.005) and 50.9%±6,26% (p<0.005), respectively (N = 8, Fig. 2).

As hemoglobin concentration was measured in every RBC lysate sample, a correlation between H_2_O_2_-induced DNA repair and hemoglobin concentration per reaction mixture was calculated. In the experiments which contained 2% RBC hemolysate the correlation coefficient was r = −0.937 (p<0.001) between H_2_O_2_-induced DNA repair (% of control) and mg hemoglobin in the reaction mixture (mean±SE = 1.90±0.114 mg). In the experiments which contained 1%, 0.6% or 0.3% ^v^/_v_ RBC hemolysate, the correlation coefficient did not reach significance.

### Effect of RBC Hemolysate on ds-DNA

**Figure 3:**
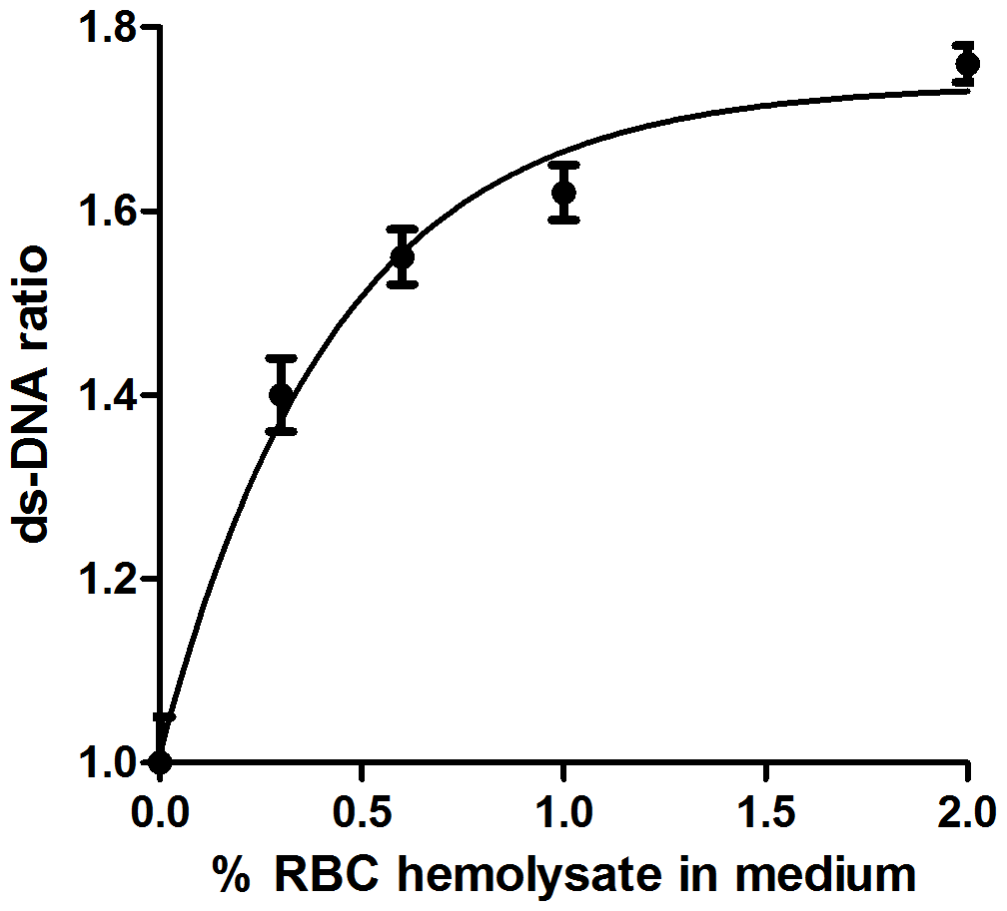
Dose-response curve of the effect of RBC hemolysate (% ^v^/_v_ in medium) on ds-DNA ratio in H_2_O_2_-stimulated PBMC. p<0.001 for the effects of the 0.3, 0.6, 1 and 2% ^v^/_v_ RBC hemolysate concentrations.

**Figure 4:**
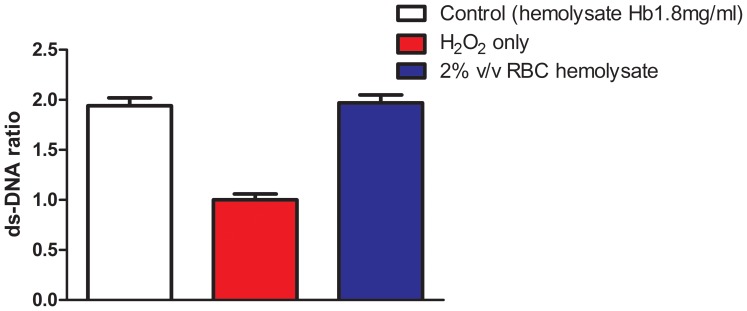
ds-DNA ratio in H_2_O_2_-stimulated PBMC: effect of purchased human hemoglobin (1.8 mg/ml) and RBC hemolysate (2% ^v^/_v_, mean hemoglobin content = 2.00±0.07 mg/ml). b vs a, p<0.001; c vs a, p<0.001; N = 8.

The reduction of H_2_O_2_-induced DNA repair by RBC hemolysate can be attributed to two mechanisms: either hemolysate inhibits the DNA repair reaction or it protects the DNA from damage induced by H_2_O_2_ and consequently reduces the need for DNA repair. In order to investigate which of the two possibilities existed in our reactions, the effect of RBC hemolysate on ds-DNA was measured. %ds-DNA was 48.0%±2.40% in PBMC excited by 200 µmol/L H_2_O_2_ without hemolysate. In the absence of H_2_O_2_, %ds-DNA was 72.1%±3.18%. This difference proved that H_2_O_2_ caused DNA damage in our reaction conditions. Without RBC hemolysate, ds-DNA ratio in H_2_O_2_-excited cells was 1.00±0.05. With the addition of 0.3%, 0.6%, 1% and 2% ^v^/_v_ RBC hemolysate to the medium, the calculated ds-DNA ratio was 1.40±0.04, 1.55±0.03; 1.62±0.03 and 1.76±0.02, respectively, p<0.001 for all concentrations (N = 10, Fig. 3). These results demonstrate that RBC hemolysate improved ds-DNA in a dose-dependent manner. In order to examine whether the hemoglobin component of the RBC hemolysate was responsible for the elevation of %ds-DNA in the presence of 200 µmol/L H_2_O_2_, several experiments were conducted with purchased human hemoglobin. At a concentration of 1.8 mg/ml reaction solution, ds-DNA ratio was significantly elevated from 1.00±0.06 to 1.94±0.08% (p<0.001, N = 8, Fig. 4). This result is similar to ds-DNA affected by 2% ^v^/_v_ RBC hemolysate, which contained 2.00 mg±0.07 mg hemoglobin/ml reaction solution.

### Correlation between H_2_O_2_-induced DNA Repair and ds-DNA

**Figure 5:**
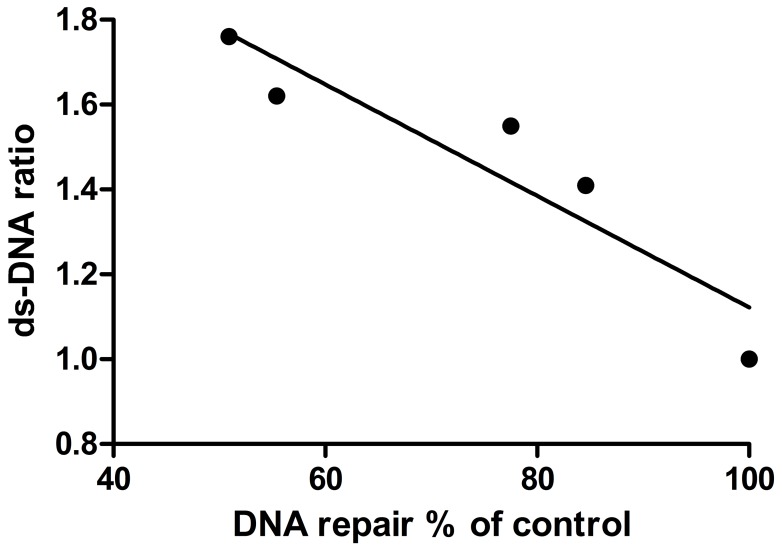
Correlation between ds-DNA ratio (control = 1.00) and DNA repair (% of control) in H_2_O_2_-stimulated PBMC, at concentrations of 0, 0.3, 0.6, 1 and 2% ^v^/_v_ RBC hemolysate. For each point, N = 8. Coefficient r = −0.925, p<0.025. Regression line: y = −0.131x +2.432.

For every concentration of RBC hemolysate in the reaction medium two results were obtained, DNA repair ability and %ds-DNA. As there were five points of % RBC hemolysate in the medium, we calculated the correlation coefficient between these two parameters. Every point represented the means of the results. The correlation coefficient between ds-DNA and DNA repair ability was r = −0.925 (p<0.025, N = 5 for 0%, 0.3%, 0.6%, 1% and 2% hemolysate in medium). Regression line was y = −0.131x +2.432 (Fig. 5). This highly significant negative correlation demonstrated that the more double strands existed in DNA, the less DNA repair reaction was induced to repair the strand breaks. These results indicated that hemolysate/hemoglobin did not reduce DNA repair by inhibiting DNA repair reaction but by elevating the amount of ds-DNA.

### Histone H2AX Phosphorylation

**Figure 6:**
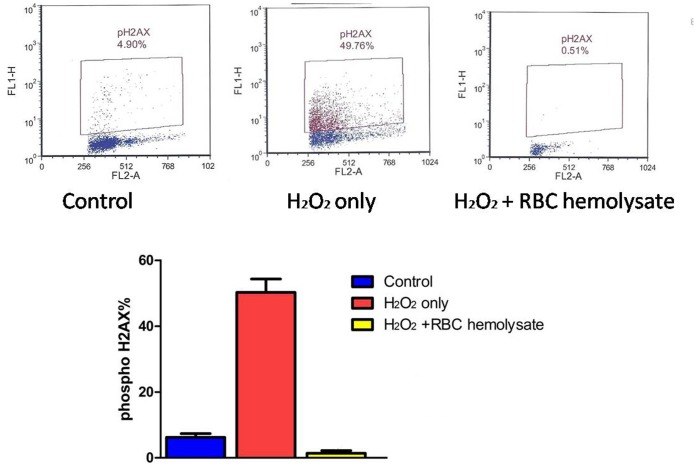
Induction of activation and H2AX phosphorylation in PBMC cells exposed to 200 µM H_2_O2 for 40 min. (a) PBMC were untreated; or (b) treated with 200 µM H_2_O_2_ for 40 min;(C) or treated with 200 µmol/L H_2_O_2_ for 40 min with hemolysate, each detected immunocytochemically, measured by laser scanning cytometry (scatterplots). The dashed skewed lines show the upper threshold level of PBMC and phosphorylated H2AX expression. % of PBMC expressing H2AX (n = 3) is shown in the graph.

As illustrated in Fig. 6, phosphorylated H2AX rose significantly from 5.96±0.61% to 45.54±3.63% following incubation of PBMC with H_2_O_2_. The addition of RBC hemolysate to the reaction with H_2_O_2_ prevented the rise in phosphorylation of histone H2AX, which was 1.03±0.27%. The Friedman two way analysis of variance showed a significant difference between all three groups (N = 3, p<0.028).

### Comet Assay

**Figure 7:**
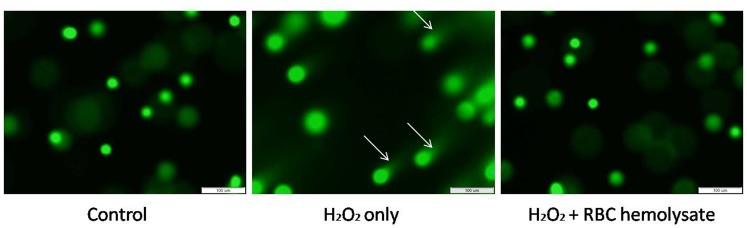
Comet assay – a representative experiment (N = 3). The left image depicts the control. The middle image depicts the effect of H_2_O_2_. The right image depicts the effect of H_2_O_2_ and RBC hemolysate.

To ascertain the effect of H_2_O_2_ on DNA, we used the comet assay. Fig. 7 is representative one of 3 assays. As can be seen, H_2_O_2_ caused DNA breaks, as expected (middle), while the addition of RBC hemolysate prevented the damage to the DNA (right).

## Discussion

In the present study we found that RBC, RBC hemolysate and hemoglobin reduced DNA repair ability in PBMC following H_2_O_2-_induced DNA breaks. The effect of the RBC hemolysate was dose-dependent. Furthermore, we observed a significant negative correlation between hemoglobin and DNA repair at the concentration of 2% ^v^/_v_ hemolysate. No correlation was seen with lower concentrations of hemolysate.

The reduction in DNA repair by RBC, RBC hemolysate and hemoglobin could be due either to their direct effect on the DNA repair reaction or to a defensive effect against H_2_O_2_-induced DNA damage with the appropriate DNA repair response. Indeed, we have previously seen that the rise in DNA damage due to oxidative stress during dialysis was accompanied by a rise in DNA repair [17]. To clarify which mechanism took place, we used the FADU method which measures % of ds-DNA, assuming that the higher % of double strands represents lesser DNA breaks and calls for less repair activity. The well-known FADU method, originally designed by Birnboim [12], was recently adapted to an automatic version [13]–[15]. This method was chosen by us [18], [19] and other laboratories [20] because of its relative simplicity and its results of clear cut expression of %ds-DNA. We found that in H_2_O_2_-induced PBMC the ds-DNA increased from a ratio of 1.00 without RBC hemolysate (equal to 48.0%±2.40% ds-DNA) to a ratio of 1.76±0.02, when 2% ^v^/_v_ RBC hemolysate was present, suggesting that RBC hemolysate inhibited DNA damage by H_2_O_2_. Furthermore, there was a significant negative correlation (r = −0.925) between DNA repair and %ds-DNA collectively. These results suggest that the reduction in DNA repair is in large part due to the decrease in DNA breaks in the presence of the RBC hemolysate. These findings were supported by the results of the comet assay and the analysis of the histone H2AX phosphorylation. Reduction in DNA breaks induced by H_2_O_2_ with the addition of RBC hemolysate to the reaction was observed by both methods. The phosphorylation of histone H2AX is an early step in recruiting and localising DNA repair proteins to the DSBs sites [17]. The reduction in phosphorylated H2AX in the presence of RBC hemolysate explains the reduction in DNA repair by the addition of RBC hemolysate. We then compared the effect of hemolysate and hemoglobin on the level of % ds-DNA following induction with H_2_O_2_ and found a similar effect. Thus, most of the hemolysate effect is probably carried out by the hemoglobin.

Our results are in accordance with the studies which found a negative correlation between hemoglobin level and DNA damage [9], and with the studies that showed protection by normal RBC [10] or fetal RBC [21] of lymphocytes against chromosomal breakage induced in lymphocytes by the alkylating agent diepoxybutane. Similar to our findings, Porto et al suggested that this effect is obtained by hemolysed RBC and more specifically by the hemoglobin [10]. They further suggested an additional protective effect of fetal RBC due to increased antioxidative activity by the increased glutathione transferase, catalase and superoxide dismutase activity in fetal RBC [21]. Furthermore, a positive correlation between total plasma antioxidant capacity and hemoglobin levels was found [9], [22]. Both total plasma antioxidant capacity and hemoglobin levels were negatively correlated with lymphocyte DNA damage [9]. Moreover, lymphocyte DNA damage was significantly higher in anemic patients compared to that in the control group [9].

DNA damage may lead to cell aging and carcinogenesis [17], [23]. Our results suggest that RBC hemolysate and hemoglobin have an important role in the preservation of DNA integrity against oxidative DNA damage. Collectively, the results of this study and other studies suggest that anemia predisposes to increased oxidative stress-induced DNA damage, whereas a higher hemoglobin level provides a better protection against such DNA damage. Thus, in addition to RBC’s main task of oxygen transport, they seem to contribute to the preservation of the DNA.
